# To Test or Not to Test: Routine Thrombophilia Diagnostic Screening of Women with Reproductive Failures

**DOI:** 10.3390/jcm12247527

**Published:** 2023-12-06

**Authors:** Urszula Wysocka, Kinga Sałacińska, Iwona Pinkier, Łukasz Kępczyński, Wojciech Ałaszewski, Lech Dudarewicz, Agnieszka Gach

**Affiliations:** Department of Genetics, Polish Mother’s Memorial Hospital Research Institute, 93-338 Lodz, Poland; kinga.salacinska@iczmp.edu.pl (K.S.); iwona.pinkier@iczmp.edu.pl (I.P.); lukasz.kepczynski@iczmp.edu.pl (Ł.K.); wojciech.alaszewski@iczmp.edu.pl (W.A.); lech.dudarewicz@iczmp.edu.pl (L.D.)

**Keywords:** recurrent pregnancy loss (RPL), thrombophilia, factor V, prothrombin

## Abstract

Background: Recurrent reproductive failure is a global health issue affecting a significant number of women. Thrombophilias have been implicated as a possible cause. Inherited thrombophilias include a single nucleotide variant on factor V Leiden and prothrombin. Objective: The aim of this study was to evaluate the association between the following single nucleotide variants: factor V Leiden (c.1601G>A), the prothrombin gene (c.*97G>A) and the reproductive failure in the Polish population. Methods: The study was conducted in a group of 545 patients with recurrent pregnancy loss, RPL (≥2 miscarriages), and in a group of 641 patients with infertility. The distribution of genotypes for the selected variants were determined by RFLP-PCR and by the real-time PCR method. Results: A variant of the *F5* gene was found in 5.14% of patients with RPL and in 6.08% of infertile women. A variant of the *F2* gene was identified in 0.73% of patients with RPL and in 2.03% of women with infertility. The frequency in the study groups did not differ from that in the general population. No association between the studied variants of the *F5* gene or the *F2* gene and the predisposition to reproductive wastage was found. Conclusions: Recommendations for routine thrombophilia testing in women with recurrent miscarriages should be revisited. The decision regarding testing should be made individually depending on additional factors indicating an increased risk of venous thromboembolism.

## 1. Introduction

**Figure 1:**
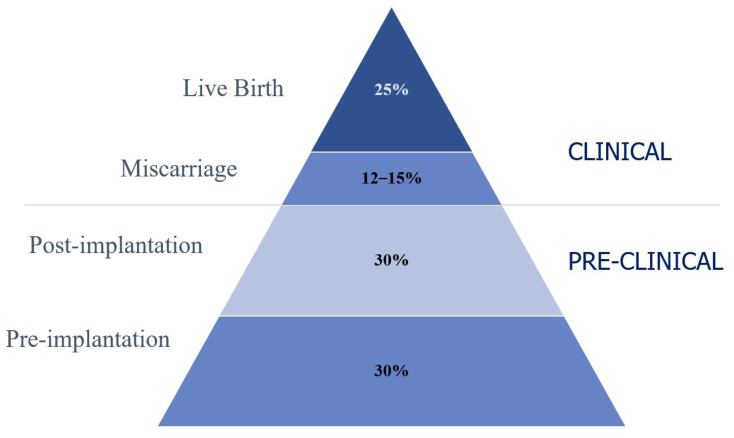
The pregnancy loss iceberg (adapted from Chard 1991 and Macklon 2002) [4,5].

The chance of having offspring depends on the normal anatomical structure and functional efficiency of the reproductive system, as well as a number of interacting processes taking place from the moment of conception, through the duration of pregnancy, and until childbirth. In humans, compared to many other species, procreation is an inefficient process, which may be due to the fact that the incidence of abnormal embryos, including those with chromosomal aberrations, is very high [1,2]. It is estimated that 70–75% of conceptions are lost prior to live birth. The majority of these losses occur prior to implantation or before a missed menstrual period, and since they are not revealed to the woman, they are termed preclinical. Thus, only around 25% of conceptions progress to a live birth [3] (Figure 1).

Reproductive failure (RF) is a global health problem. These phenomena have social and demographic implications. Reproductive failures are also a source of extreme stress and psychological problems for a woman and her partner.

Infertility affects approximately 10–15% of couples of reproductive age worldwide [6]. Infertility is defined as the failure to achieve pregnancy after 12 months of regular unprotected sexual intercourse. Approximately 85% of infertile couples have an identifiable cause. The most common causes of infertility are ovulatory dysfunction, male factor infertility, and tubal disease [7]. Infertility in women is extremely heterogeneous, which reflects the complex interaction of numerous developmental, hormonal, environmental, and genetic factors [8].

The role of thrombophilia in infertility is still controversial. Based mainly on the results of observational studies [9], some authors have suggested that thrombophilias could be involved in infertility and, although there is no clear evidence regarding the positive impact of screening and treatment on these patients [10,11,12,13], it is used in routine clinical practice. Recurrent pregnancy loss (RPL) is a disease distinct from infertility, defined by the spontaneous loss of two or more pregnancies [14,15]. The etiology of the disease comprises different factors, such as autoimmune diseases (20%), endocrinological disorders (17–20%), uterine alterations (10–15%), genetic factors such as chromosome abnormalities in the parents (2–5%) and infections (0.5–5%) [16]. Among other etiologies, the failure of implantation can result in infertility [17]. Nevertheless, approximately 50% of RPL cases remain unexplained and defined as idiopathic [1,18].

The etiopathogenesis of reproductive failure is varied. Thrombophilias have been implicated as a possible cause.

Thrombophilia is a group of inherited or acquired coagulation disorders. Congenital thrombophilia is defined as a genetic predisposition to venous thromboembolism (VTE), usually triggered by the absence or alteration of a functional protein in the coagulation cascade. Combined with the hypercoagulable state of pregnancy, thrombophilia has the potential to induce placental thrombosis and cause placental insufficiency with subsequent obstetrical complications [19]. On the other hand, from an evolutionary perspective, variants of genes associated with thrombophilia that lower the risk of hemorrhage may have conferred a survival advantage to the species. Historically, lethal exsanguinations and severe infections have been two major causes of maternal death. The high prevalence of a relatively common variant of factor V Leiden (c.1601G>A) in the general population suggests that it may actually serve the reproduction of humanity. This variant reduces the risk of blood loss and profuse hemorrhage due to childbirth and improves hemoglobin status [19,20].

Inherited thrombophilias include a single nucleotide variant on the prothrombin *F2* (c.*97G>A; G20210A) and factor V Leiden *F5* (c.1601G>A; G1691A) genes. The population frequency of the heterozygous variant c.*97G>A *F2* gene ranges from 2 to 6% and the heterozygous variant c.1601G>A *F5* gene from 3 to 7% [21,22].

Both variants on the *F2* and factor V Leiden genes occur most often in the heterozygous form separately or combined. Patients with both homozygous variants are very rare in the general population [23]. Patients with either the heterozygous variants on *F2* or *F5* genes are at a mild risk of thrombosis, and 4.9 and 3.8 times more likely to have a first blood clot, respectively. However, if the patient is a carrier of both heterozygous variants, then the risk becomes higher and increases by up to 20 times. The deficiencies of antithrombin, protein C, protein S, plasminogen and dysfibrinogenemia are less common among inherited thrombophilias. Thrombophilia is a multifactorial condition and only the mutual interactions between the environment and genes may lead to the development of clinical manifestations [21,24]. The role of hereditary thrombophilia in reproductive failure (RF) is strongly debatable [10].

The acquired tendency to develop thrombophilia and VTE is related to a lot of clinical or environmental–hypercoagulable states. The most common factors leading to the acquirement of thrombophilia are laparoscopic surgery, arthroscopic knee surgery, age, obesity, smoking, and immobility due to sitting and bedrest lasting more than three days [21]. Other thrombophilia and VTE risk factors are malignancy, pregnancy, oral contraceptives or hormone replacement therapy [21].

Screening for thrombophilia in obstetric practice remains controversial because of the limited evidence of a true causal relationship with pregnancy complications and the lack of a proven effective intervention [25]. Furthermore, the diagnosis of thrombophilia based on coagulation tests rather than genotypes to identify single nucleotide variants (SNV) may lack precision and may be affected by the hemostatic changes in pregnancy and the postpartum period [26].

The aim of this study was to assess the validity of routine screening for inherited thrombophilia in women with reproductive failure in the Polish population.

## 2. Materials and Methods

### 2.1. Sample Collection

Case groups were enrolled between September 2013 and March 2022 in the Department of Genetics at the Polish Mother’s Memorial Hospital Research Institute in Łódź. All members of the study groups were Caucasians and residents of Poland, with no immunological diseases, weight disorders [obesity body mass index (BMI) < 30 kg/m^2^], hypertension, diabetes or coagulation disorders.

The study was positively evaluated by the Bioethics Committee at the Polish Mother’s Memorial Hospital Research Institute in Łódź. All participants were informed of the study protocol and completed a consent form before participating in the study.

This population-based study was conducted on 1186 Polish women (aged 19–46 years) who were divided into two groups. Group 1 included 545 women with recurrent pregnancy loss (RPL), with a history of two or more (even seven) consecutive spontaneous abortions (mean age of 32.42 ± 4.88 years old). Group 2 consisted of 641 women with infertility (mean age of 32.78 ± 4.45 years old).

Peripheral venous blood samples (3–5 mL) from patients were collected into EDTA-coated vacutainers. Genomic DNA was isolated from peripheral blood leukocytes by standard procedures using a commercially available kit, no. 101, for DNA isolation from blood, and a MagCore HF16 Plus automat (RBC Bioscience, New Taipei City, Taiwan). The concentration and quality of the DNA were examined via optical density in a spectrophotometer NanoDrop 2000 (ThermoFisher Scientific, Waltham, MA, USA).

### 2.2. Sample Examination and Statistical Analysis

Depending on the time of the analysis, samples were tested by RFLP-PCR (using restriction enzyme HindIII) or by the real-time PCR method. The tests were carried out with commercial kits according to the manufacturer’s recommendations. Detection was based on the real-time PCR method with the use of fluorescently labelled probes and specifically on the principle of allelic discrimination. The study was performed on a CFX OPUS 96 Dx instrument (Bio-Rad, Hercules, CA, USA).

To assess whether the empirical data fulfills the Hardy–Weinberg equilibrium, a chi-squared test with Yates continuity correction was used, and a *p* < 0.05 level was treated as significant. Non-significant results were treated as following the Hardy–Weinberg equilibrium.

## 3. Results

A single nucleotide variant c.*97G>A of *F2* gene (GA genotype) was identified in 4 (0.7%) patients with RPL and in 13 (2%) women with infertility. A variant c.1601G>A of the *F5* gene (GA genotypes) was found in 28 (5.1%) patients with RPL and in 39 (6.1%) infertile women (Table 1). A co-incidence of heterozygous genotype GA of variant *F2* and *F5* genes was observed in only one woman with spontaneous miscarriage. Genotype AA for the variants of *F2* and *F5* genes was not observed in any of the examined patients.

The frequency of allele A of the *F2* gene was 0.4% and 1% in patients with RPL and in women with infertility, respectively. Compared to the described European populations, the frequency of this variant is relatively low (about 6%) [21]. With regard to allele A of the *F5* gene, it was present with a frequency of 2.6% and 3% for both study groups. This result is even lower than the distribution of this allele in the populations of North, Central and South-Central European countries with a value of about 5%.

The distribution of genotypes and alleles of the studied variants was similar for both groups (for the variant *F2* gene, *p* = 0.997 for patients with RPL and *p* = 0.985 for women with infertility, and for the variant *F5* gene, *p* = 0.944 and *p* = 0.921 respectively). The results were considered statistically insignificant. The frequency in the study groups did not differ from that in the general population. Due to the high frequency of tested variants in the general population, the results obtained in this study were related to information from available databases. The minor allele frequency (MAF) in the Genome Aggregation Database (gnomAD) is 0.02472 for c.1601G>A and 0.01290 for c.*97G>A. No association between the studied variants c.*97G>A *F2* gene or c.1601G>A *F5* gene and the predisposition to reproductive failure was found.

## 4. Discussion

The prothrombin gene (*F2*), also known as coagulation factor II, is located on chromosome 11p11.2. The transition nucleotide G (guanine) to A (adenine) at *97 in the 3′-untranslated (3′UTR) region of the *F2* gene (variant c.*97G>A, rs1799963; c.20210G>A) causes increased prothrombin levels and an increased risk of VTE.

Women with thrombophilia are at a higher risk of VTE during pregnancy. In several studies, c.*97G>A heterozygotes had a 3- to 15-fold higher risk of pregnancy-associated VTE than pregnant women without inherited thrombophilia [27,28]. Heterozygous women without a family history of VTE have a lower thrombotic risk than women with prothrombin thrombophilia and a family history of VTE. Although c.*97G>A heterozygosity increases the relative risk of pregnancy-associated VTE, the absolute risk in asymptomatic heterozygotes is low in the absence of other predisposing factors with an estimated probability in the range of 1:200 to 1:300 pregnancies [27].

Women homozygous for c.*97G>A or compound heterozygous for c.*97G>A and factor V Leiden have a higher relative risk of pregnancy-associated VTE, but the absolute risk is less well defined [28]. The probability of VTE during pregnancy and the puerperium is lower in compound heterozygous women younger than 35 years of age (5.5%) than in older women (8.2%) [29].

An analysis of the correlations of the c.*97G>A prothrombin gene variant has been already performed in the Polish population.

We did not find an association between the studied variant of the *F2* gene and reproductive failure. These observations were similar to the results presented by Skrzypczak et al. and Pasińska et al. [30,31]. However, not all studies are consistent with the results of the present study. Wolski et al., Barlik et al. and Ślęzak et al. indicated that this variant was associated with reproductive failure [22,32,33].

The discrepancies regarding the effect of the explored variants on the risk of reproductive failure may be due to the fact of population differences, group size, inclusion criteria and the effect of other immunological, genetic or environmental factors.

The *F5* (coagulation Factor V) gene is located on chromosome 1q24.2. The transition of G (guanine) to A (adenine) in 1601 nucleotide position in exon 10 causes changes in the protein chain: the substitution of arginine for glutamine (R506Q). This variant identified as c.1601G>A (rs6025; c.1691G>A) leads to the resistance of coagulation factor V to proteolytic inactivation by the activated protein C (APC), which is consequently related to a predisposition to thrombosis.

Normal pregnancy is associated with a 5- to 10-fold increased risk of developing VTE. Women heterozygous for the Leiden variant have a five to eight times greater risk of pregnancy-related VTE than women without the variant [28,29,34]. The risk is higher in women from families with a history of thrombosis and in women older than 34 years of age.

While heterozygosity for the Leiden variant increases the relative risk of pregnancy-associated VTE, the absolute risk is low in the absence of other predisposing factors. VTE is estimated to occur in 1% of pregnancies in women who are Leiden-variant heterozygotes. The absolute risk increases to 3% in those with a positive family history of VTE [35,36].

In women homozygous for the Leiden variant the relative risk is increased 17- to 34-fold [28,29]. The absolute risk of developing pregnancy-related VTE is estimated at 2.2–4.8% of pregnancies. The risk is higher (14%) in homozygotes with a positive family history and in those older than 34 years of age [29,34].

Women who are compound heterozygotes for the Leiden variant and the c.*97G>A *F2* variant are reported to have an 8- to 47-fold increased relative risk of pregnancy-related VTE [29,37]. The probability of VTE during pregnancy and the puerperium is lower (5.5%) in doubly heterozygous women younger than 35 years of age than in older women (8.2%) [29].

The findings of this study are in keeping with the reports of Wolski et al., Ślęzak et al. and Bałajewicz-Nowak et al. [22,32,38]. In contrast, Skrzypczak et al. and Pasińska et al. showed that the variant c.1601G>A was significantly associated with an increased susceptibility to reproductive failure [30,31].

Wawrusiewicz-Kurylonek et al.’s study showed that the pathogenic variants in the *F2* and *F5* genes were observed in 2.7% and 7% of the analyzed group of the Polish population, respectively [21]. Adler et al. showed the incidence value of the variant c.1601G>A of the *F5* gene at 2.0% [39].

Our study showed that the frequency of the studied variants in the *F2* and *F5* genes in the studied groups of patients does not differ from the frequency of these variants in women in the general population.

It should be emphasized that individuals who carry either or both variants of the c.*97G>A of *F2* gene and c.1601G>A of *F5* gene may never develop VTE symptoms due to the multifactorial nature of this disease. Only a combination of various risk factors along with genetic factors such as surgery, hospitalization with prolonged immobilization or estrogen therapy can lead to the provoking of a clinical manifestation of thrombophilia [21]. Up to a certain age, carriers of pathogenic variants remain asymptomatic because the risk of VTE increases with age [40].

The treatment strategies for thrombophilia depend on the underlying hypercoagulable state and the clinical presentation. In the treatment of thrombophilia, it is important to prevent the first episode of thrombosis (primary prevention) and subsequent episodes (secondary prevention). The mainstay of therapy for thrombophilia is anticoagulation with either warfarin, low molecular weight heparin (LMWH), direct Xa inhibitors, or direct thrombin inhibitors. The majority of patients with congenital thrombophilia should be on long-term direct oral anticoagulants (DOAC) therapy which is currently the preferred therapeutic option mainly due to the lower risk of major bleeding (12).

In the meantime, given the lack of clear benefits from treatment or any impact on prognosis, screening for heritable thrombophilia in the situation of pregnancy loss is not warranted [26]. The association between thrombophilia and pregnancy complications is contributory rather than causative [41]. The current evidence base for inherited thrombophilias and pregnancy complications is largely retrospective, with heterogeneity in classifications and populations, leading to conflicting results. At present, universal thrombophilia screening is not recommended, and recommendations for which clinical subgroups should undergo screening vary nationally and internationally [42]. This discordance between guidelines reflects the paucity of evidence of cost-effectiveness including therapeutic efficacy [26].

It is debated whether inherited thrombophilias result in adverse pregnancy outcomes, and the results of studies are mixed on whether there is an association with RPL. Screening for thrombophilia should entail a comprehensive assessment of the patient’s prothrombotic state, not just a laboratory test [43].

The European Society of Human Reproduction and Embryology (ESHRE) suggests against screening for hereditary thrombophilia for women with RPL unless in the context of research or for women with additional risk factors for thrombophilia [44,45].

The British Fertility Society, in their recommendations, concluded that testing for hereditary thrombophilia is not indicated in recurrent implantation failure (RIF) [19,46].

According to the American Society for Reproductive Medicine (ASRM), screening for inherited thrombophilias (specifically, factor V Leiden and prothrombin gene mutations, protein C, protein S, and antithrombin deficiencies) may be clinically justified when a patient has a personal history of venous thromboembolism in the setting of a non-recurrent risk factor (such as surgery) or a first-degree relative with a known or suspected high-risk thrombophilia. Although an association between hereditary thrombophilias and fetal loss has been suggested [47,48], prospective cohort studies have failed to confirm this [49,50]. Routine testing of women with RPL for inherited thrombophilias is not currently recommended [51,52].

Following recent recommendations, routine testing for inherited and/or acquired thrombophilia in patients with reproductive failure is not recommended, although it could be useful in some instances to identify infertile women who could benefit from anticoagulant therapy [13].

## 5. Conclusions

The obtained results suggest that the analyzed variants of *F2* and *F5* genes in the Polish population have little predictive value in diagnostics for women with reproductive failure. In our opinion, the decision regarding testing should be made individually depending on additional factors indicating an increased risk of venous thromboembolism. Recommendations for routine thrombophilia testing in women with recurrent failures should be revisited.

## Figures and Tables

**Table 1:** Alleles and genotypes distribution in the study groups.

rs Number Genotype/Allele	Study Group	Patients with RPL	Patients with Infertility
	N = 1186	N = 545	N = 641
*F2* (rs1799963)			
GG	1169 (98.6%)	541 (99.3%)	628 (98%)
GA	17 (1.4%)	4 (0.7%)	13 (2%)
AA	-	-	-
G	2355 (99.3%)	1086 (99.6%)	1269 (99%)
A	17(0.7%)	4 (0.4%)	13 (1%)
*F5* (rs6025)			
GG	1119 (94.4%)	517 (94.9%)	602 (93.9%)
GA	67 (5.6%)	28 (5.1%)	39 (6.1%)
AA	-	-	-
G	2305 (97.2%)	1062 (97.4%)	1243 (97%)
A	67 (2.8%)	28 (2.6%)	39 (3%)

## Data Availability

The datasets presented in this study are available from the corresponding author. The data are not publicly available due to the individual’s private information.
